# Medical Institute

**Published:** 1843-12

**Authors:** 


### MEDICAL INSTITUTE.

The class at the Medical Institute this winter, is the largest out one that has yet assembled within its walls.

